# Emerging evidence of neutrophil extracellular traps in essential hypertension and target organ damage: pathophysiological insights and therapeutic implications

**DOI:** 10.1038/s41440-026-02724-3

**Published:** 2026-07-14

**Authors:** Anastasia Malliora, Efstratios Gavriilidis, Antonios Lazaridis, Christina Antoniadou, Agapi Chionidou, Vasilios Kotsis, Panagiotis Skendros, Eugenia Gkaliagkousi

**Affiliations:** 1 3rd Department of Internal Medicine, Papageorgiou General Hospital, Aristotle University of Thessaloniki, Thessaloniki, Greece; 2 First Department of Internal Medicine, University Hospital of Alexandroupolis, Democritus University of Thrace, Alexandroupolis, Greece

**Keywords:** hypertension, hypertension-mediated organ damage, immunity, neutrophil extracellular traps, NET inhibitors

## Abstract

Essential hypertension (EH) remains the leading modifiable risk factor for cardiovascular morbidity and mortality, yet even optimally treated patients carry residual cardiovascular risk. Emerging evidence suggests that chronic subclinical inflammation contributes to the pathogenesis of EH, leading to target organ damage. Neutrophil extracellular traps (NETs), which represent networks of decondensed chromatin carrying neutrophil proteins, have recently gained attention as potent drivers of a harmful thromboinflammatory milieu. While NETs are established contributors to atherosclerotic cardiovascular disease, including ischemic stroke, coronary artery disease and heart failure, their role in hypertension has only recently begun to be elucidated. Preclinical studies demonstrate that NETosis may promote endothelial dysfunction, vascular smooth muscle cell proliferation, and blood pressure elevation, while inhibition of NETs formation attenuates these processes. Clinical data, though limited, indicate that untreated hypertensives exhibit higher circulating NETs, linked to enhanced thrombogenicity, adverse vascular remodeling, and potentially to hypertension-mediated organ damage. Furthermore, therapeutic strategies targeting NETs, ranging from conventional immunomodulatory therapies to biologic agents, are under investigation. This review summarizes current knowledge on the role of NETs in the pathophysiology of EH and related cardiovascular diseases, while discussing potential anti-NETotic effects of currently available pharmacological agents.

**Figure:**
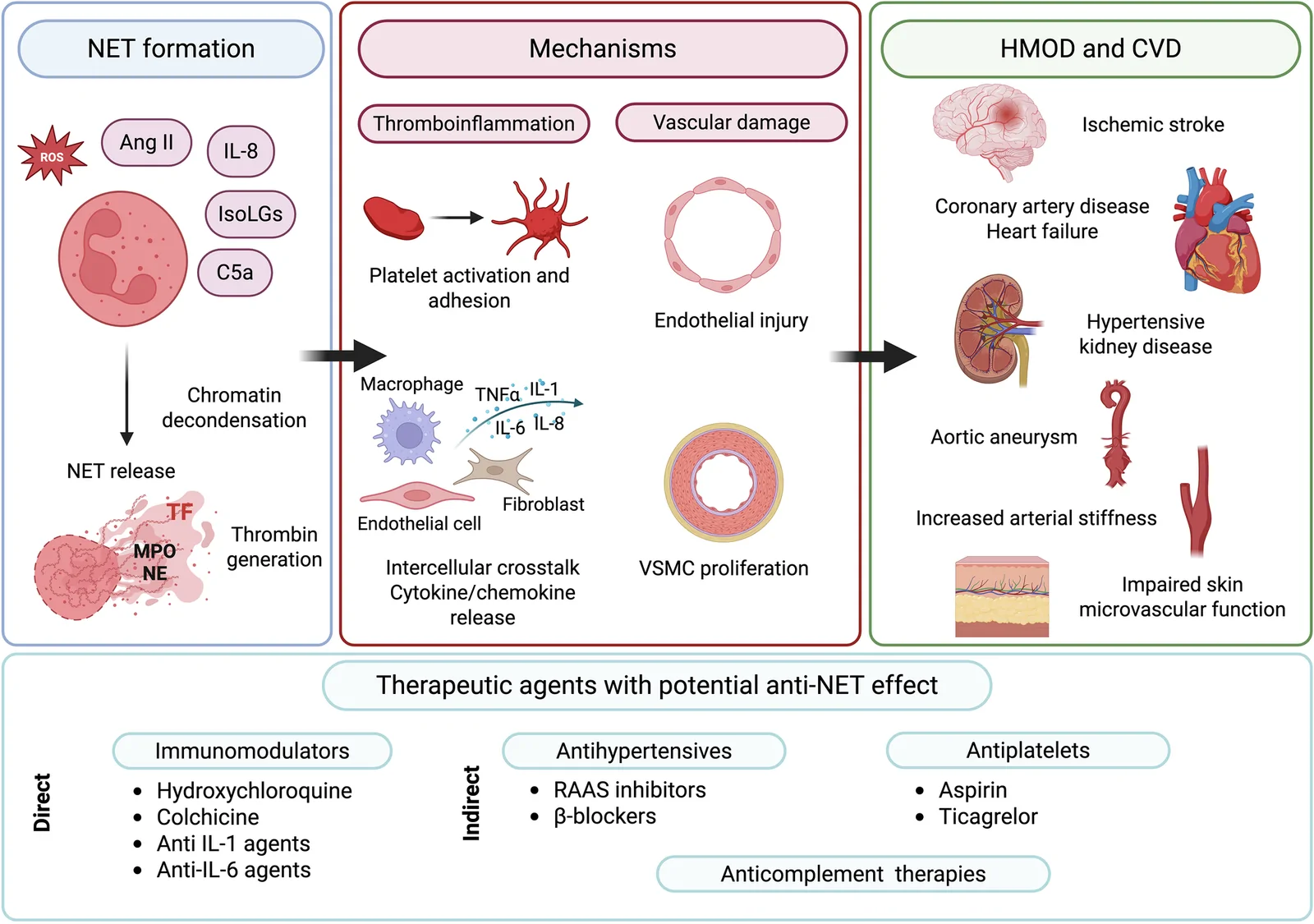
Hypertensive stimuli promote neutrophil extracellular traps (NETs) formation, leading to thromboinflammation, endothelial injury and vascular damage. These processes are involved in the occurrence of hypertension-mediated organ damage and cardiovascular disease, while sustaining elevated blood pressure. Targeting NET formation may interrupt this vicious cycle and reduce residual cardiovascular risk in hypertension. Ang II, angiotensin II; C5a, compliment component 5a; CVD, cardiovascular disease; HMOD, hypertension-mediated organ damage; IL, interleukin; IsoLGs, isolevuglandins; MPO, myeloperoxidase; NE, neutrophil elastase; NET, neutrophil extracellular traps; RAAS, renin-angiotensin-aldosterone system; ROS, reactive oxygen species; TF, tissue factor; TNFα, tumor necrosis factor α; VSMC, vascular smooth muscle cells

Hypertensive stimuli promote neutrophil extracellular traps (NETs) formation, leading to thromboinflammation, endothelial injury and vascular damage. These processes are involved in the occurrence of hypertension-mediated organ damage and cardiovascular disease, while sustaining elevated blood pressure. Targeting NET formation may interrupt this vicious cycle and reduce residual cardiovascular risk in hypertension. Ang II, angiotensin II; C5a, compliment component 5a; CVD, cardiovascular disease; HMOD, hypertension-mediated organ damage; IL, interleukin; IsoLGs, isolevuglandins; MPO, myeloperoxidase; NE, neutrophil elastase; NET, neutrophil extracellular traps; RAAS, renin-angiotensin-aldosterone system; ROS, reactive oxygen species; TF, tissue factor; TNFα, tumor necrosis factor α; VSMC, vascular smooth muscle cells

## Abstract (web summary)

These authors contributed equally: Anastasia Malliora, Efstratios Gavriilidis

## Introduction

Essential hypertension (EH) is the most important modifiable risk factor contributing to cardiovascular morbidity and mortality worldwide and it is responsible for almost half of all cases of stroke, heart failure, myocardial infarction, and kidney disease [1]. Hence, the more effective control of blood pressure (BP) in hypertensive patients has become a clear necessity. Interestingly, it has been demonstrated that even well-controlled hypertensives experience a significant residual cardiovascular risk [1], even under combined antihypertensive therapy targeting two main mechanisms involved in the pathophysiology of hypertension, namely the sympathetic nervous system (SNS) and the renin-angiotensin-aldosterone system (RAAS). These observations imply the contribution of other pathways mediating organ damage and, thus, cardiovascular risk to the already complex hypertensive environment.

Towards this direction, over the past decade experimental and clinical studies have highlighted the role of chronic subclinical inflammation, propagated by a dysregulated immune response involving both innate and adaptive immunity, in pathways orchestrating the pathogenesis of hypertension [1, 2]. An ever-increasing amount of evidence has corroborated the role of innate immunity in fueling an augmented inflammatory response in hypertension. In this context, a significant amount of bioactive molecules (i.e., nucleic acids, fatty acids, heat shock proteins [HSPs], and high-mobility group box-1 [HMGB1]), all collectively termed alarmins or damage-associated molecular patterns (DAMPs), may be released in the hypertensive microenvironment upon damage to the vascular wall [3].

Cellular components of innate immunity include monocytes, macrophages, dendritic cells, and granulocytes, the latter being the majority among all immune effectors. For several decades, perturbations of neutrophil counts and their properties have been documented in experimental models of hypertension. Lately, growing interest has been focused on extracellular structures of decondensed chromatin networks expelled by neutrophils, named neutrophil extracellular traps (NETs) [4, 5]. NETs constitute one of the main inflammatory mechanisms employed by neutrophils in response to a wide range of stimuli [6] and were initially described as a protective mechanism with microbicidal effect [4]. However, it has later been shown that dysregulated or excessive NET formation, as well as the release of NETs enriched with pathogenic bioactive molecules, can sustain a thromboinflammatory environment that contributes to tissue damage. Consequently, it has been recognized that NETs are actively involved in the pathophysiology of a variety of diseases with an inflammatory background, such as autoimmune and cardiovascular diseases (CVD) [7, 8].

Despite the fact that the contribution of immunity to the pathogenesis of hypertension has already been identified [9], the available data regarding the role of NETs in hypertension are limited. In this review we aimed to describe the emerging role of NETs and their contribution to the pathophysiology of hypertension and hypertension-associated organ damage (HMOD). Additionally, we discuss the potential modulatory effects of currently available pharmacological agents on NET formation and activity.

## Induction, formation and composition of NETs

NETs, first described in 2004 by Brinkmann et al. [4], are extracellular networks composed of decondensed chromatin decorated with proteins from neutrophil granules and cytoplasm, such as myeloperoxidase (MPO), neutrophil elastase (NE) and citrullinated histones (CitH3). A complex, yet not fully elucidated cascade results in NET formation with reactive oxygen species (ROS) playing a major role. ROS stimulate the translocation of MPO and NE from the neutrophil granules to the neutrophil nucleus, where these enzymes modify histones and facilitate extensive chromatin decondensation [5]. In response to several inflammatory stimuli, neutrophils release NETs, in an attempt to trap and kill extracellular microbes, thus acting as a protective antibacterial mechanism [4, 10]. Beyond their role in immune defense, NETs also serve as carriers of various bioactive molecules, including cytokines and coagulation factors. Importantly, these molecules acquire significant activity when embedded in NETs, thereby enabling them to orchestrate biological processes, such as modulation of inflammatory pathways, induction of thrombus formation, and fibrotic processes. This indicates that NETs may serve as dynamic mediators capable of shaping immune responses, vascular damage, and tissue remodeling across a wide range of pathological inflammatory conditions [11–13], including CVD [7, 8].

## Methods for the assessment of NETosis

A variety of techniques are used to detect NET formation, including immunofluorescence microscopy, immunohistochemistry, flowcytometry and assays measuring circulating NET components such as MPO-DNA complexes, CitH3, nucleosomes, and extracellular/free DNA. Among these, MPO-DNA and CitH3 are the most commonly used circulating biomarkers due to their accessibility and relative specificity. However, limitations exist: CitH3 mainly reflects PAD4-dependent NETosis and may not capture all NET formation pathways, while MPO-DNA assays may show reduced specificity in plasma due to overlap with other inflammatory processes [14]. Therefore, combining multiple markers and techniques may improve reliability whereas in tissue samples, immunofluorescence microscopy remains the reference method for NET detection [14–16].

## General mechanistic background linking NETs to endothelial injury, thromboinflammation and atherosclerosis

### Endothelial injury and thromboinflammation

In recent years, NETs have been recognized as central mediators of thromboinflammation, a process in which the bidirectional and dynamic interplay between inflammation and thrombosis amplifies both inflammatory and thrombotic phenotypes. The role of thromboinflammation has been highlighted during the COVID-19 pandemic where patients with aberrant inflammation leading to respiratory failure were characterized by an exceedingly high risk of thrombosis and cardiovascular complications [17]. NETs provide a link between innate immune responses, endothelial dysfunction, and activation of the coagulation system [18, 19].

From a mechanistic point of view, several features of NETs appear to directly contribute to prothrombotic processes. NETs may induce endothelial injury, thereby promoting platelet adhesion and activation. In parallel, NETs are decorated with bioactive tissue factor (TF) and other coagulation factors, contributing to thrombin generation. Thrombin further activates platelets, leading to the release of inorganic polyphosphates (polyP) that amplify NET formation within the vasculature. These NETs then provide a structural scaffold for fibrin deposition and thrombus stabilization. Concurrently, crosstalk between NETs and endothelial cells induces the overexpression of adhesion molecules, such as ICAM-1/VCAM-1, further facilitating neutrophil recruitment and adhesion. NET-associated proteases, including NE, degrade and inactivate endogenous anticoagulants such as antithrombin and tissue factor pathway inhibitor (TFPI), collectively amplifying the prothrombotic environment [20–25].

Moreover, NETs act as immunothrombotic signaling platforms. Their scaffold, composed of extracellular DNA and histones, engages pattern recognition receptors (PRRs), including Toll-like receptors (TLRs) on platelets, and other cell populations such as macrophages, endothelial cells, and fibroblasts. This interaction activates downstream signaling pathways such as NF-κB and the NLRP3 inflammasome, resulting in the transcription and release of proinflammatory cytokines (e.g., interleukin [IL]-1β, IL-6, and tumor necrosis factor α [TNFα]) that sustain vascular inflammation. Additionally, chemokine production by stromal cells, notably IL-8 (CXCL8), promotes further neutrophil recruitment and NET release, thereby establishing self-amplifying positive feedback loops that perpetuate thromboinflammation [12, 19, 26–28].

Importantly, NETs engage in a reciprocal and reinforcing relationship with the complement system during thromboinflammatory processes. Activated complement components stimulate NET formation, while NETs themselves carry bioactive molecules that regulate complement convertases, thereby sustaining cycles of inflammation and thrombosis [18, 23, 29]. More specifically, C5a has been demonstrated to be a potent inducer of thromboinflammation by activating neutrophils via C5a receptors and prompting TF expression on NETs [29, 30]. Moreover, other complement components, such as C3a, have been associated with IL-8-driven thromboinflammation [31]. In addition, NETs have been found to carry key components of the complement alternate pathway, including complement factor P, B, and properdin, further augmenting the feedback loops between NETs and the complement cascade [32].

### Endothelial injury and atherosclerosis

The contribution of NETosis to atherosclerosis is linked to endothelial cell injury, thromboinflammation, and lipid accumulation. More specifically, endothelial erosion leads to the release of TF containing microparticles which activate the coagulation cascade. Furthermore, the subsequent exposure of the subendothelial matrix promotes neutrophil adhesion and activation. Finally, activated neutrophils release TF-rich NETs, while this phenomenon is further exacerbated by ROS [33–35]. Moreover, neutrophils release chemokines and activate distinct cell populations, such as platelets and monocytes/macrophages [36, 37], while through NETs they promote the proliferation of vascular smooth muscle cells (VSMCs). In turn, within the plaque, both VSMCs and macrophages absorb lipids and transform into foam cells, contributing respectively to the fibrous-cap remodeling and formation of the lipid core [37, 38].

Evidence from experimental studies in human and mouse plaques have revealed the first signs of NETs’ involvement in atherosclerosis by showing an abundance of neutrophils and NETs in complex plaques with thrombosis or rupture [39]. More specifically, immunohistochemistry analysis of plaque segments from 30 patients with acute myocardial infarction demonstrated increased accumulation of NETs in all types of complicated, but not intact, plaques. Remarkably, large concentrations of neutrophils and NETs were also observed in the nearby perivascular tissue of complicated, but not intact, plaques. Neutrophils and NETs were more commonly found in fresh, non-organized thrombi and in ongoing intraplaque hemorrhages [40]. Another study demonstrated the presence of NETs in thrombi retrieved from patients with ischemic stroke and myocardial infarction. NETs were more abundant in stroke thrombi, although this difference may reflect the earlier and more intensive antiplatelet/anticoagulant therapy administered in myocardial infarction [41]. The discovery that histone H4-mediated membrane lysis of VSMCs promotes inflammation and plaque destabilization, supported the above findings [42].

Beyond the key role of complement in thromboinflammation, activated complement components have been also implicated in the initiation and progression of atherogenesis, where they promote endothelial dysfunction, lipid accumulation, and plaque inflammation [43]. In this context, complement activation establishes additional feedback loops with NETs in the pathogenesis of atherosclerosis.

## NETs in essential hypertension

Hypertension is a well-established risk factor for cardiovascular complications. Despite a growing amount of data indicating the contribution of NETs to critical pathogenetic pathways of cardiovascular disease such as inflammation, thrombosis, and atherosclerosis, so far, evidence regarding their role in the hypertensive microenvironment is limited. More specifically, few data exist regarding the mechanisms linking NETs with elevated BP and, therefore, HMOD. Of note, recent evidence has shown that NETs are detected in target organs of hypertensive patients, a finding that could imply the contribution of NETs to hypertensive vascular damage. We hereby present all the currently available evidence from experimental and clinical studies on the role of NETs in EH, including their levels, the association with BP, and their contribution to vascular and organ damage both at micro- and macrovascular level (Table 1).

**Table 1:** (a) Preclinical and (b) clinical studies regarding the role of NETs in EH

Study	Year	NETosis marker	Identification method	Population	N	Main findings
**a. Preclinical studies**						
Krishnan et al. [46]	2024	CitH3	Immunofluorescence	*Padi4*^*−/−*^ mice	NA	(1) PAD4 deficiency decreases aortic inflammation, enhances endothelial function, improves vascular relaxation, and attenuates hypertension. (2) Hypertensive stretch stimuli significantly increase NETosis and accumulation of neutrophil CitH3. (3) CitH3 built up disrupts endothelial function, directly contributing to NETosis-induced endothelial cell dysfunction, vascular dysfunction, and enhanced inflammatory cytokine expression.
Fang et al. [47]	2022	MPO, CitH3	Western blotting and immunofluorescence	SHR and BALB/c mice	NA	(1) CitH3 expression increases in arterial walls of SHRs indicating a link between NETosis and hypertension. (2) Blood pressure increased in PMA injected mice and MPO and CitH3 expression was elevated indicating a link between NET formation and hypertension. (3) NET formation increases VSMC proliferation via TK1. (4) VSMC proliferation is upregulated in VSMCs cultured with NETs exosomes.
Krishnan et al. [48]	2022	MPO, NE, dsDNA, CitH3	ELISA, flow cytometry and immunofluorescence	Human blood samples (isolated neutrophils) and C57BL/6 mice	human samples: 15 mice: NA	(1) The specific isoLG scavenger 2HOBA reduces neutrophil genes and affects neutrophil migration. (2) CitH3 and MPO increase in Ang II treated mice but decrease in 2HOBA treated mice indicating IsoLG scavengers prevent NETosis. (3) In isolated human neutrophils, isoLGs’ accumulation during NETosis is reversed by 3HOBA treatment. (4) During NETosis, IsoLGs contribute to nucleosome assembly and nuclear expansion.
**b. Clinical studies**						
Malliora et al. [15]	2025	MPO-DNA complexes, CitH3	ELISA	UHTs and NTs	139 (99 UHTs / 40 NTs)	(1) MPO-DNA complexes and CitH3 were elevated in UHTs in comparison to NTs. (2) The majority of office BP and ABPM parameters were associated with MPO-DNA complexes. (3) Among all BP parameters, 24 h peripheral systolic BP was the strongest predictor of hypertension-related NETosis. (4) There was a strong correlation between MPO-DNA complexes’ levels and markers of vascular damage, including carotid PWV and skin microcirculation indices.
Li et al. [44]	2022	cf-DNA, nucleosomes, MPO–DNA complexes, NE	ELISA and immunofluorescence	EH patients and normotensive controls	218 (168 patients / 50 controls)	(1) Moderate or severe EH patients have increased NETosis markers in comparison with those with mild EH and controls. (2) Inflammatory cytokines enhance NET formation in EH patients directly and indirectly by activating platelets. (3) NETs increase the levels of coagulation factors resulting in decreased clotting time in EH patients and this effect can be diminished by DNase I. (4) ECs are activated to promote coagulation by NETs.
Chrysanthopoulou et al. [16]	2021	MPO/DNA complexes, CitH3	ELISA and immunofluorescence	UHTs and health controls	81 (55 patients / 26 controls)	(1) In comparison to healthy individuals, patients with EH have increased NETosis markers and EH plasma can promote NETosis in control neutrophils. (2) Neutrophils stimulated by EH patients’ plasma release NETs carrying bioactive TF which affect TAT activity. (3) AngII promotes NETosis via a ROS/augophagy manner. (4) In ARB-treated EH patients, NET formation is diminished along with decreased TF and TAT activity.
Hofbauer T et al. [45]	2017	Exclusively extracellular DNA released from cells with disrupted cellular membranes	Sytox® Green Nucleic Acid Stain (fluorescent dye)	Patients with coronary artery disease and healthy controls	NA	(1) NETosis was promoted by ionomycin and the level of NET formation depended on its dosage. (2) Treatment with AngII increased the level of NET formation by ionomycin. (3) The effect of AngII on NETosis was attenuated by the ARB losartan and the NADPH oxidase inhibitor DPI.

*2HOBA* 2 hydroxybenzylamine, *ABPM* ambulatory blood pressure monitoring, *AngII* angiotensin II, *ARB* angiotensin II receptor blocker, *BP* blood pressure, *CitH3* citrullinated histone 3, *DPI* diphenyleneiodonium chloride, *ECs* endothelial cells, *EH* essential hypertension, *ELISA* enzyme linked immunosorbent assay, *Et2HOBA* ethyl-2-hydroxybenzylamine, *IsoLG* isolevuglandin, *MPO* myeloperoxidase, *NA* non-available, *NADPH* nicotinamide adenine dinucleotide phosphate, *NETs* neutrophil extracellular traps, *NTs* normotensive controls, *PAD4* protein-arginine deiminase-4, *Padi4*^*−/−*^ mice lacking protein-arginine deiminase-4, *PMA* phorbol-12-myristate-13-acetate, *PWV* pulse wave velocity, *ROS* reactive oxygen species, *SHR* spontaneously hypertensive rat, *TAT* thrombin-antithrombin, *TF* tissue factor, *TK1* thymidine kinase 1, *UHTs* untreated newly diagnosed patients with essential hypertension, *VSMC* vascular smooth muscle cell

### Evidence of increased levels of NETs

Recent studies performed in hypertensive patients have shown significantly higher plasma levels of circulating NETs in these patients compared to normotensive controls. More specifically, MPO/DNA complexes and CitH3 plasma levels were elevated in untreated, newly diagnosed hypertensive patients, free from overt CVD compared to age- and sex-matched healthy controls [15, 16]. Notably, antihypertensive treatment with a RAAS inhibitor targeting angiotensin II (AngII) signaling was associated with a reduction in NET levels. Although these findings derive from a relatively small cohort, they suggest a potential link between RAAS activation and NET formation implying a possible interplay between RAAS and innate immunity activation from the early stages of EH.

### Evidence of association of NETs with BP

Beyond the differences between hypertensive and normotensive individuals, circulating NETs correlate with BP levels. In particular, a recent publication examined the association of NETs burden with BP in a group of 99 newly diagnosed, treatment-naïve hypertensive patients, free from overt CVD who were compared with 40 normotensive controls. In univariate analysis, office BP and the majority of ambulatory BP parameters, both peripheral and central, were significantly correlated with plasma levels of NETs, whereas hypertension per se emerged as the strongest independent predictor of NETosis, even after adjustment for confounding factors [15]. In addition, Li et al. documented a clear association between plasma levels of NETs and the severity of hypertension. Of interest, patients with moderate to severe EH had higher levels of NETs as compared to normotensive controls and patients with mild EH [44]. Similarly, Hofbauer et al. showed that NETs in patients with coronary artery disease (CAD) (not only hypertensives) exhibit a positive correlation with peripheral BP. Interestingly, the above association did not change after accounting for their baseline treatment, taking into consideration that all patients were receiving medications with blood pressure lowering effect [45]. Overall, the above findings highlight a potential association between NETs and BP regulation, however causality cannot be inferred due to the cross-sectional design of the available studies.

### Evidence of association of NETs with thrombosis, endothelial and vascular dysfunction in hypertension

A recent study in hypertensive patients demonstrated that circulating NETs exhibit potent thrombogenic activity [16]. In fact, plasma from untreated EH patients was shown to induce the release of NETs from control neutrophils that are enriched with bioactive TF, thereby promoting the activation of the TF/thrombin axis. This was further supported by significant elevated levels of thrombin-antithrombin (TAT) complexes in patients’ plasma compared with controls. Notably, circulating NET burden, as reflected by CitH3 levels, positively correlated with TAT activity, underscoring the link between NET formation and thrombin generation. Mechanistically, EH plasma was able to induce TF expression in control neutrophils and drive the formation of TF-bearing NETs, which were functionally active in promoting coagulation. Importantly, disruption of NETosis with DNase I or neutralization of TF significantly attenuated TAT generation and TF activity, highlighting the central role of NET-associated TF in mediating thromboinflammation in EH [16].

In addition, another study showed that NETs from patients with moderate to severe EH shortened clotting time and enhanced fibrin and thrombin generation, overall suggesting a highly thrombogenic potential. Furthermore, it was shown that DNase I, an enzyme responsible for the breakdown of NETs, can significantly mitigate these procoagulant effects by around 70%. Finally, endothelial cells developed a procoagulant phenotype as a result of the potent cytotoxic effects induced by high concentrations of NETs [44]. However, in this study, neutrophil/NETs studies at the tissue level were not performed.

Limited experimental observations have also started to highlight the role of NETs as drivers of vascular dysfunction in hypertension. In particular, mice lacking protein-arginine deiminase-4 (Padi4^−/−^), an enzyme essential for NETosis, exhibit blunted responses to AngII, which are associated with decreased aortic inflammation, enhanced endothelial function, and improved vascular relaxation. In the same study, coculture of neutrophils with endothelial cells and simultaneous exposure to hypertensive stretch stimuli led to significant increase in NETosis and accumulation of neutrophil CitH3. Subsequently, it was shown that CitH3 accumulation disrupts endothelial function, directly contributing to NETosis-induced endothelial cell dysfunction, enhanced inflammatory cytokine expression, and vascular dysfunction [46]. Collectively, the above findings identify the important contribution of NETosis to the impairment of endothelial function in hypertension.

Moreover, another experimental study by Fang et al. has documented that the burden of NETs accumulated in the arterial wall of spontaneously hypertensive rats (SHRs) is significantly increased compared to that of normotensive Wistar–Kyoto (WKY) rats. At the same time, it was observed that induction of NETosis by injecting a potent ROS inducer named phorbol-12-myristate-13-acetate (PMA), enhanced MPO and CitH3 upregulation in the arterial wall, which was further associated with increased BP. Additionally, NETs were found to promote VSMC proliferation through thymidine kinase 1 (TK1), whose expression was found to positively correlate with BP [47]. Moreover, another experimental study has shown that isolevuglandins (IsoLGs), which are by-products of lipid peroxidation formed in the presence of ROS, independently promote NET formation in isolated human neutrophils by driving chromatin decondensation during NETosis. At the same time, it was demonstrated that treatment of hypertensive rats with an isoLG scavenger, namely 2-hydroxybenzylamine (2HOBA), prevents renal and aortic NET accumulation, and peripheral neutrophil migration [48].

### Evidence of association of NETs with HMOD

A few data exist regarding the association of NETs with organ damage in the hypertensive environment. Moreover, most evidence is derived by studies with relatively small cohorts and cross-sectional designs.

At microvascular level, NETs burden has been associated with alterations in skin microvascular bed. More specifically, a study in 99 newly diagnosed, untreated hypertensive patients and 40 normotensive controls showed that plasma levels of NETs are negatively correlated with several markers of skin microvascular function including baseline to peak flux and cutaneous vascular conductance increase. This finding could imply a possible relationship between NETosis and very early microvascular injury considering that skin microvascular changes are among the earliest vascular abnormalities observed in EH [15]. At macrovascular level, NETs have been associated with arterial stiffness, a key surrogate marker of large artery damage. More specifically, the levels of circulating NET markers, such as MPO-DNA complexes, have been shown to independently predict carotid artery stiffness in hypertensive patients. In addition, NET components have been detected in core tissues affected by hypertension, including kidney and the vascular wall. Indeed, NETotic neutrophils and deposition of NETs remnants have been observed in fibrotic areas of kidney biopsies from patients with hypertensive nephropathy but not in biopsies from normotensive patients, indicating a potential profibrotic role of NETosis in hypertension. Likewise, NETs remnants have been detected in tissue sections from abdominal aortic aneurysms obtained from hypertensive patients [16].

**Fig. 1:**
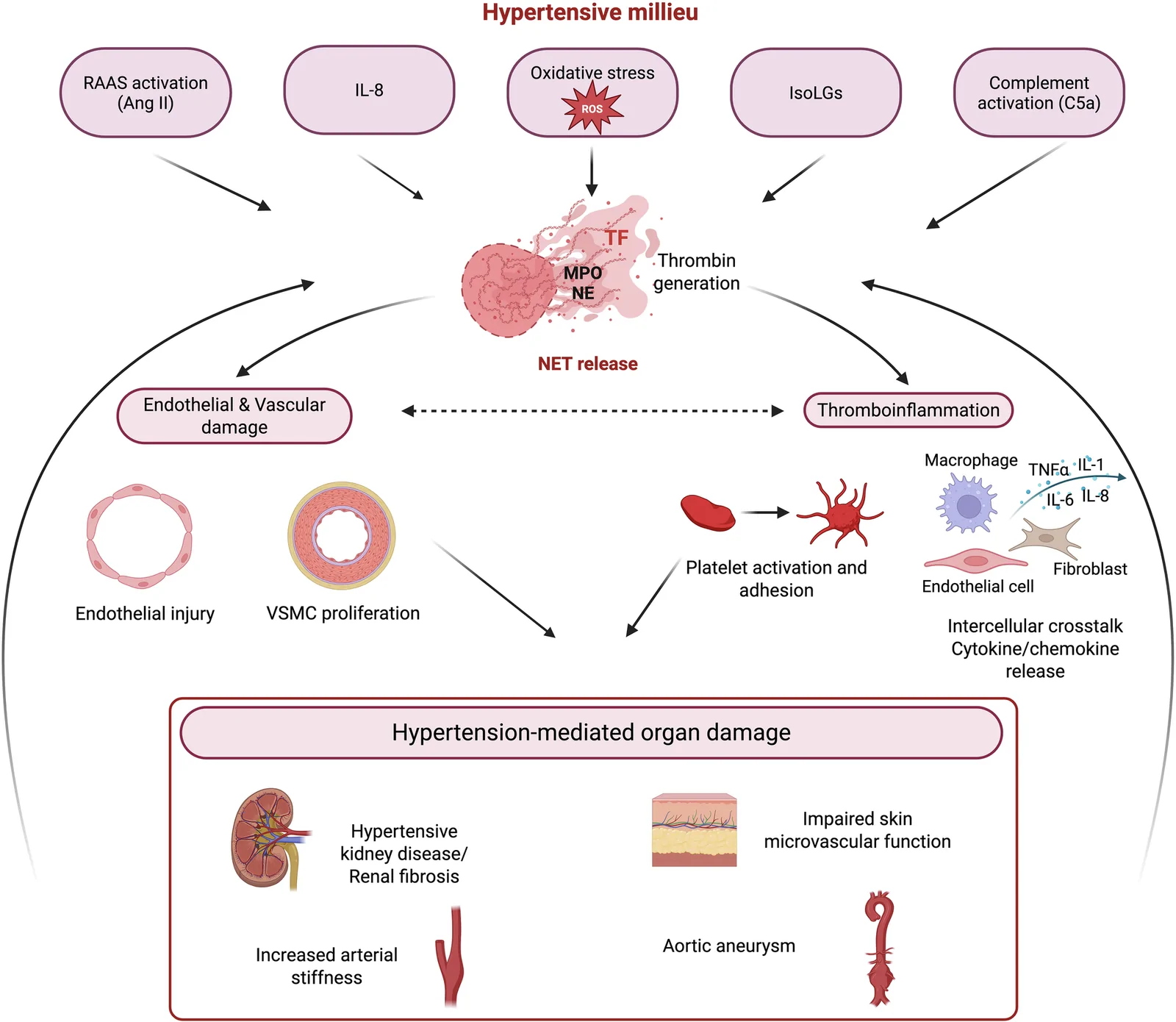
Schematic overview of neutrophil extracellular traps formation and its contribution to thromboinflamation, endothelial and vascular damage, and consequently to hypertension-mediated organ damage. Ang II angiotensin II, C5a compliment component 5a, IL interleukin, IsoLGs isolevuglandins, MPO myeloperoxidase, NE neutrophil elastase, NET neutrophil extracellular traps, ROS reactive oxygen species, TF tissue factor, TNFα tumor necrosis factor α, VSMC vascular smooth muscle cells

To sum up, the evidence suggesting a definite role for NETosis in hypertension is yet limited and stems mainly from experimental and observational studies, underscoring the need for larger prospective and longitudinal investigations to enable more robust interpretation. However, it has been already documented that RAAS activation and oxidative stress, which form core pathogenetic mechanisms of EH, are able to drive NETosis and the release of NETs with a highly thrombogenic and inflammatory potential. In turn, it has been shown that NETs may further promote endothelial and vascular dysfunction in hypertension. From a clinical perspective of view, ongoing clinical data show that levels of NETs are increased in hypertensive patients and are strongly associated with BP. Collectively, based on the above, it could be assumed that NETs could take their share in the pathophysiologic continuum linking inflammation, thrombosis, endothelial and vascular dysfunction in hypertension (Fig. 1).

## NETs in established CVD

As outlined above, NETs have been increasingly implicated in the pathophysiology of EH, a well-established risk factor for CVD, through their role in thromboinflammation, endothelial dysfunction, and vascular injury. Importantly, EH is the strongest modifiable risk factor for stroke and one of the most significant risk factors for coronary heart disease and heart failure. A growing body of evidence supports the involvement of NETs in these hypertension-related cardiovascular entities, where similar mechanisms are thought to underlie disease development and progression.

### Ischemic stroke

NETs have been identified in human brain ischemic lesions [49]. Moreover, increased circulating NET levels have been found in patients with ischemic stroke in comparison to healthy controls and have been correlated with stroke functional outcomes [50, 51] and all-cause mortality at one-year follow-up [52]. Importantly, NETs burden has been associated with both cardioembolic and atherosclerotic stroke, supporting a role for NETs in thrombus initiation, stabilization, and resistance to therapeutic fibrinolysis [41]. Interestingly, ex vivo thrombolysis of patient thrombi was found to be more successful when adding DNase I, an agent that dismantles NETs compared with standard tissue plasminogen activator (t-PA) treatment [51]. Additionally, NETs interact with platelet microvesicles, exacerbating thrombosis and brain injury and contributing to t-PA resistance [53, 54]. Finally, experimental studies have shown that neutrophils-derived cell-free DNA contributes to plaque destabilization after stroke, while DNase I may reduce recurrent events [55].

### Coronary artery disease

NETs contribute to acute coronary syndromes primarily through their involvement in thrombogenesis and atherosclerosis. In line with this, it has been demonstrated that neutrophil accumulation in coronary thrombi [56, 57] and independently predict mortality [58–60]. NETosis markers are increased in patients with severe CAD compared to healthy controls, and correlate with the number of atherosclerotic coronary artery vessels and the severity of luminal stenosis [61]. During acute ST-elevation myocardial infarction (STEMI), thrombin-activated platelets and neutrophils interact at the site of plaque rupture and result in local NET formation and active TF delivery [35]. Importantly, in STEMI, NETs burden positively correlates with infarct size and negatively correlates with resolution of ST-segment on electrocardiogram [62], while CitH3-DNA levels predict major adverse cardiac events (MACEs) and mortality, especially when combined with conventional inflammatory markers, such as C-reactive protein (CRP) [63].

### Heart failure

Inflammation is a core mechanism implicated in heart failure (HF), and neutrophils systematically infiltrate the dysfunctional cardiac tissue [64]. Elevated levels of NETs have been demonstrated in patients with HF, suggesting a potential relationship between NETosis and disease severity [65, 66]. More specifically, NETs may promote myocardial injury, inflammation, and fibrosis, thus actively contributing to ventricular remodeling and dysfunction [67]. NETs may also serve as carriers of bioactive peptides, including matrix metalloproteinases (MMPs) involved in tissue remodeling [28, 68, 69]. Moreover, CitH3 plasma levels were recently correlated with levels of brain natriuretic peptide in patients with end-stage HF and decreased after left ventricular assist device implantation. Of interest, CitH3 levels were significantly decreased after the operative procedure [70], while myocardial NET deposition in idiopathic dilated cardiomyopathy was negatively associated with left ventricular ejection fraction [71]. Notably, data specifically addressing NETosis across HF phenotypes remain limited.

## Therapeutic agents with potential anti-NET effect

The experimental and clinical findings described above highlight the potential role of NETs in EH and CVD as drivers of vascular damage and suggest that targeting NETosis could represent a promising future therapeutic strategy. Notwithstanding, most of the currently available therapeutic strategies do not exhibit a direct suppressing effect on NETs while, at the same time, the majority of available evidence is derived from clinical studies not specific to hypertension. In this section, we summarize all the pharmacological agents that may have a direct or indirect impact on NET formation. Table 2 provides additional information about their level of evidence.

**Table 2:** Therapeutic agents with potential anti-NETotic effect and impact in CVD: mechanisms and level of evidence

Therapeutic agent	Proposed mechanism of action	Evidence for NETosis inhibition	Evidence in CVD
**a. Anti-inflammatory agents**			
Hydroxychloroquine	Direct: Inhibits PAD4, TLR-9, and decreases IL-8 and ROS	In vitro & animal studies [72–75]	Small observational studies [77, 78]
Colchicine	Direct: Disrupts microtubules and thus inhibits MPO/NE translocation	In vitro studies [79, 80]	3 RCTs, Guidelines conditional recommendation [81, 83–85]
Anakinra (IL-1Ra)	Direct: Blocks IL-1-induced NET formation and decreases intracellular calcium mobilization, disrupts IL-1β/DNA complexes	In vitro & small observational studies [86, 87, 92]	Animal, small observational study [88, 89]
Canakinumab (IL-1β inhibitor)	Direct: Suppresses NETosis via IL-1β inhibition, disrupts IL-1β/DNA complexes	In vitro & small observational studies [86, 87, 92]	1 RCT & secondary analysis [90, 91]
Tocilizumab (sIL-6Ra)	Direct: Suppresses NETosis via IL-6 blockade	In vitro studies [92]	2 RCTs & secondary analysis [93–95]
**b. Antihypertensive agents**			
ACEi/ARBs	Indirect: Inhibits AngII-mediated ROS NETosis	Animal and small observational studies [16, 97]	Guidelines recommendation
β-blockers	Indirect: Inhibits neutrophil activation and recruitment	Animal and small observational studies [98, 99]	Guidelines recommendation
**c. Antiplatelets**			
Aspirin	Indirect: Inhibits NF-κB	In vitro and animal studies [101]	Guidelines recommendation
Ticagrelor	Indirect: Inhibits platelet-derived polyP	In vitro studies [102, 103]	Guidelines recommendation
**d. Anticomplement therapies**			
C3/C5 inhibitors	Indirect: Inhibits complement-mediated NETosis	In vitro and animal studies [23, 30, 105, 106]	Small observational studies [107, 108]

*ACEi* angiotensin converting enzyme inhibitor, *AngII* angiotensin II, *ARB* angiotensin type receptor blocker, *CVD* cardiovascular disease, *IL* interleukin, *IL-1Ra* interleukin-1 receptor antagonist, *MPO* myeloperoxidase, *NE* neutrophil elastase, *NETs* neutrophil extracellular traps, *PAD4* protein arginine deiminase 4, *polyP* polyphosphates, *RCTs* randomized controlled trials, *ROS* reactive oxygen species, *sIL-6RA* soluble interleukin-6 receptor antagonist, *TLR* toll-like receptors

### Therapies exerting a direct impact on NETs

#### Anti-inflammatory agents

##### Hydroxychloroquine

Hydroxychloroquine, an antimalarial drug mainly used in autoimmune rheumatic diseases, can inhibit NET formation through inhibition of PAD4 and TLR-9 expression, and decreased production of IL-8 and ROS [72–75]. Sagris et al. suggested that hydroxychloroquine may inhibit atherosclerotic plaque progression in ApoE knockout mice, through the inhibition of autophagy and NET formation [76]. In a clinical study by Baker et al. including rheumatoid arthritis patients, treatment with hydroxychloroquine was associated with a significant decrease in both systolic BP (SBP) and diastolic BP (DBP), and a higher probability of achieving optimal BP control at 6 months [77]. Similarly, in systematic lupus erythematosus patients, hydroxychloroquine led to small, albeit significant decline in mean BP and SBP, along with better BP control within 3-months of follow-up [78]. Whether these BP-lowering effects can be attributed solely to hydroxychloroquine’s inhibitory action on NETs formation or to other drug-driven anti-inflammatory mechanisms, remains to be further elucidated.

##### Colchicine

Colchicine is a key treatment for various autoinflammatory diseases, such as familial Mediterranean fever (FMF) and Behcet’s disease. It inhibits the polymerization of tubulin in the inflammasome and ultimately reduces the production of pro-inflammatory cytokines, such as IL-1 [79, 80]. The drug may also act as a direct NETs inhibitor by binding to microtubules and disrupting the trafficking of NE and MPO from cytoplasmic granules to the nucleus, an essential step in NET formation. Interestingly, despite its well-described anti-NETotic/anti-inflammatory effects, colchicine is characterized as a BP-neutral medication by large randomized controlled trials [81]. However, there is evidence that it acutely increases β-adrenoceptor and nitric oxide-mediated vasodilation in humans, suggesting that it might exert effects on vascular stiffness and resistance, beyond inflammation [82]. Indeed, when administered at low doses, colchicine has demonstrated significant reductions in major cardiovascular events among patients with atherosclerotic myocardial infarction [81, 83, 84], making it the first anti-inflammatory agent to gain approval by the Food and Drug Administration (FDA) for cardiovascular risk reduction in patients with established CVD. Importantly, its use has now been incorporated into the most recent American Heart Association (AHA)/American College of Cardiology (ACC) clinical guidelines, which state that low-dose colchicine may be considered as an adjunct for secondary prevention in atherosclerotic CVD (ASCVD) patients [85].

##### Anti-IL-1 agents

Anakinra is a recombinant human IL-1 receptor antagonist approved for the treatment of autoinflammatory diseases, such as adult-onset Still’s disease and FMF, which are characterized by aberrant release of IL-1-bearing NETs during attacks. It exerts a potent effect on pathogenetic NET-associated inflammation by inhibiting soluble IL-1, a cytokine capable of inducing NET formation, as well as by directly blocking IL-1 bound to NET structures during autoinflammatory flares [86, 87]. Anakinra has been found to reduce BP and renal fibrosis in pre-clinical one-kidney/salt-induced hypertension mouse models [88]. Furthermore, clinical studies in obese adults have reported that treatment with anakinra may exert a BP lowering effect, an outcome associated with increased vasodilation mediated by elevated levels of angiotensin 1–7 peptide [89]. Another anti IL-1 agent, namely canakinumab, a humanized monoclonal antibody against IL-1β, was effective to reduce cardiovascular events in patients with atherosclerotic disease in the CANTOS trial, while secondary analysis of the study did not reveal any effects on BP [90, 91].

##### Anti-IL-6 agents

IL-6 is a potent inducer of NETosis, while in turn, NETs can be a source of IL-6 in the sites of inflammation [92]. ASSAIL-MI was the first clinical trial to assess the role of tocilizumab, a humanized anti-IL-6 receptor monoclonal antibody, in patients with CVD. Particularly, in patients with acute STEMI, tocilizumab was found to increase the myocardial salvage compared to placebo [93]. In a sub-analysis of the trial, Kindberg et al. found that patients treated with tocilizumab exhibited a reduction in NETs, suggesting that its beneficial effects on myocardial ischemia may be mediated through the suppression of NETosis [94]. In another phase 2 trial, ziltivekimab, a humanized monoclonal antibody against the IL-6 ligand, reduced biomarkers of inflammation and thrombosis relevant to atherosclerosis (RESCUE trial) [95], while the subsequent, ongoing ZEUS trial aims to further explore its effects in patients with atherosclerotic CVD, chronic kidney disease, and elevated high-sensitivity CRP (hsCRP) [96]. Further mechanistic studies are warranted to investigate the beneficial effects of anti-IL-6 agents in relation to NETosis.

### Therapies exerting an indirect impact on NETs

#### Antihypertensive agents

Components of RAAS may influence NET generation. In the study by Chrysanthopoulou et al., AngII induced NET formation in human neutrophils in vitro through ROS- and autophagy-dependent manners, while their preincubation with irbesartan, an AngII type 1 (AT1) receptor blocker (ARB), significantly reduced the effect. Accordingly, in the clinical cohort of the same study, previously hypertensive patients receiving ARB therapy and achieving normotension exhibited significantly lower circulating NET markers [16]. Moreover, an experimental study by Khan et al. reported that transgenic mice, engineered to overexpress angiotensin converting enzyme (ACE) specifically in neutrophils, exhibited increased ROS production and NET release in response to an infectious stimulus. Notably, pretreatment with ramipril for a few days attenuated this effect [97].

The role of β-blockers has also been explored. In a randomized study of mechanically ventilated patients with COVID-19-related acute respiratory distress syndrome, metoprolol administration yielded lower neutrophil counts and NET markers (CitH3 and MPO) in patients’ bronchoalveolar lavage [98]. Further results regarding the effect of metoprolol derive from a murine study employing the drug in distinct murine models. In a myocardial ischemia-reperfusion setting, metoprolol reduced neutrophil recruitment after myocardial infarction, while in a model of lipopolysaccharide-induced acute lung injury, metoprolol administration attenuated the presence of NETs in the lung tissue [99].

Given that these agents are central to the management of EH/HMOD and some CVDs, one could hypothesize that part of their benefit may stem from their NETosis-limiting effect. While the evidence supporting this link is promising, more data are needed to elucidate their exact effect on NET formation.

#### Antiplatelet agents

Aspirin is widely used as an antiplatelet therapy for secondary prevention in patients with established CVD [100]. In vitro studies showed that pretreatment of human neutrophils with high concentrations of aspirin reduced Phorbol myristate acetate (PMA)- or TNFα-induced NETosis through NF-κΒ inhibition. In the same study employing a mouse model of peritonitis, aspirin pretreatment resulted in reduced neutrophil chemotaxis and lower NET presence in peritoneum [101].

Ticagrelor, a P2Y12 inhibitor, has also been explored in in vitro models of NETosis. PolyP, released by activated platelets, induces NETosis [102]. Interestingly, ticagrelor attenuates polyP-mediated NET formation, despite neutrophils lacking P2Y12 receptors, thus indicating a mechanism independent of its classical antiplatelet action and suggesting additional mechanisms [103].

#### Anticomplement therapies

The interplay between the complement system and NETosis has been associated with endothelial dysfunction, microvascular inflammation, and vascular remodeling processes highly implicated in the pathogenesis of hypertension [17, 104]. Inhibition of C3a or C5a has been linked with lower neutrophil adhesion and reduced generation of TF-bearing NETs [105]. At preclinical level, deficiency of the C3a and C5a receptors has been shown to prevent AngII-induced hypertension in mice [106]. Moreover, C3 was found to be overexpressed in mesenchymal tissues of SHRs, while C3 knockout was able to abolish salt-sensitive hypertension [105]. At clinical level elevated serum C3 levels were associated with prehypertension in a cohort of 7820 patients [107]. Furthermore, the C5 inhibitor eculizumab has proven effective in the treatment of hypertensive emergencies and in improving renal survival in patients with atypical hemolytic uremic syndrome (aHUS) [108, 109]. However, treatment with eculizumab has also been reported, in some cases, to be associated with mild increases in BP [109] [https://ec.europa.eu/health/documents/community-register/2012/20120413122190/anx_122190_en.pdf].

Overall, there is increasing evidence that several of the pharmacological agents with an immunomodulatory function that are currently used, appear to modulate NET formation through direct or indirect mechanisms. However, most of the data come from studies in autoimmune, inflammatory diseases, while direct clinical evidence of anti-NETotic therapies in EH is presently absent. Further research is needed to clarify whether part of the therapeutic effect of currently available antihypertensive agents or other agents with an impact on BP could be explained by a direct anti-NETotic immunomodulatory effect.

## Conclusions and future perspectives

NETosis has been implicated in the pathophysiology of several CVDs, including ischemic stroke, CAD, and heart failure, contributing to inflammation, thrombosis and atherosclerosis. Over the last few years, accumulating evidence from experimental and clinical studies has uncovered the potential role of NETosis in the pathophysiology of EH and its associated organ damage. Notably, NETs have been found to promote endothelial dysfunction, vascular inflammation, and thrombosis, all core mechanisms implicated in the pathogenesis of HMOD. Although current findings highlight a link between NETs burden and BP, available human data remain limited and largely observational. Future studies are required in order to clarify and further explore the role of NETosis in hypertension, aiming to provide new therapeutic approaches that could offer incremental benefit beyond conventional antihypertensive therapies.
